# Structural and Functional Analysis of Human SOD1 in Amyotrophic Lateral Sclerosis

**DOI:** 10.1371/journal.pone.0081979

**Published:** 2013-12-02

**Authors:** Lorenna Giannini Alves Moreira, Livia Costa Pereira, Priscila Ramalho Drummond, Joelma Freire De Mesquita

**Affiliations:** Bioinformatics and Computational Biology Group, Department of Genetics and Molecular Biology, Federal University of Rio de Janeiro State (UNIRIO), Rio de Janeiro, Brazil; Institute of Health Science, China

## Abstract

Amyotrophic lateral sclerosis (ALS) is a fatal neurodegenerative disease with familial inheritance (fALS) in 5% to 10% of cases; 25% of those are caused by mutations in the superoxide dismutase 1 (SOD1) protein. More than 100 mutations in the SOD1 gene have been associated with fALS, altering the geometry of the active site, protein folding and the interaction between monomers. We performed a functional analysis of non-synonymous single nucleotide polymorphisms (nsSNPs) in 124 fALS SOD1 mutants. Eleven different algorithms were used to estimate the functional impact of the replacement of one amino acid on protein structure: SNPs&GO, PolyPhen-2, SNAP, PMUT, Sift, PhD-SNP, nsSNPAnalyzer, TANGO, WALTZ, LIMBO and FoldX. For the structural analysis, theoretical models of 124 SNPs of SOD1 were created by comparative modeling using the MHOLline workflow, which includes Modeller and Procheck. Models were aligned with the native protein by the TM-align algorithm. A human-curated database was developed using the server side include in Java, JMOL. The results of this functional analysis indicate that the majority of the 124 natural mutants are harmful to the protein structure and thus corroborate the correlation between the reported mutations and fALS. In the structural analysis, all models showed conformational changes when compared to wild-type SOD1, and the degree of structural alignment varied between them. The SOD1 database converge structural and functional analyses of SOD1; it is a vast resource for the molecular analysis of amyotrophic lateral sclerosis, which allows the user to expand his knowledge on the molecular basis of the disease. The SOD1 database is available at http://bioinfogroup.com/database.

## Introduction

Amyotrophic lateral sclerosis (ALS) is a fatal neurodegenerative disorder characterized by the adult onset of progressive dysfunction and loss of upper motor neurons in the motor cortex and lower motor neurons in the brainstem, spinal cord, and their associated tracts. ALS has a worldwide annual incidence of approximately 1.5 to 2 cases/100,000 and a prevalence of 6-8 cases/100,000 [1]. Age is an important predictive factor for the occurrence of the ALS, which is more prevalent in patients between 55 and 75 years old [2]. 

Most cases of ALS are sporadic; only 5-10% have genetic origin (familial Amyotrophic Lateral Sclerosis - fALS), and only approximately 20% of the fALS cases have mutations in Cu/Zn superoxide dismutase (SOD1) [3]. The X-ray crystal structures of CuZn SOD proteins from many species have been solved, predominantly in the fully metallated state, and the structure is highly conserved [4]. The mechanisms by which protein misfolding and aggregation induce cytotoxicity in neurodegenerative disorders are not yet established. For several of these disorders, e.g., Alzheimer’s and Huntington’s disease, the precursor proteins are structurally flexible and thus completely free to adopt pathogenic structures [5]. The protein SOD1 is a stable and perfectly soluble metalloenzyme that must first be locally or globally unfolded to acquire pathological function [6]. Consistent with this requirement, a remarkable property of the mutations associated with ALS on SOD1 is the decrease in the protein stability [7,8]. Currently, over 120 mutations in the SOD1 gene related to fALS have been described [1]. 

The mutations occur all over the protein structure: at the active site, at the β sheet and at the monomer interface [9]. Although the biophysical properties among the mutant enzymes vary greatly, the mutants cause exactly the same disease. The mutations change the active site geometry, the folding of SOD1 and the interaction between the monomers [10]. The mutation A4V on SOD1 is considered the most severe mutation linked to fALS, resulting in patient death only 11 months after the beginning of the symptoms [4]. The mutations that are located at the bonding region of the metallic ions are responsible for great alterations in the bonding capacity of the metallic ions and in the SOD1 activity. In contrast, the mutations that are found spread in other protein regions are called “wild-type like” for having similar characteristics to the wild-type enzyme [11]. 

The human next-generation sequencing and genome-wide association study (GWAS) projects generate millions of previously unknown single nucleotide variations (SNVs). Each newly sequenced genome reveals an average 300,000 new SNPs [12]. One of the main interests in human genome research is to discover whether specific nsSNPs affect human health. 

Several computational methods are available to predict when a mutation is disease related, starting from the protein sequence and/or protein multiple sequence alignments. The methods are based on the following: (1) sequence homology, (2) empirical rules, (3) structural criteria, (4) artificial neural networks, (5) decision trees, (6) random forests, and (7) support vector machines (SVMs). Evolutionary information that is encoded in the sequence profile is the most important piece of information for improving predictive performance, as indicated by the results of several predictors described in the literature [13].

We have recently performed a structural modeling and *in silico* analysis of human superoxide dismutase 2 (SOD2) [14]. In this study, we collected the natural variants of SOD1 for *in silico* analysis, which can determine whether these variants influence the protein’s three-dimensional structure or stability. Possible effects of the missense variants on protein function could be inferred using bioinformatics tools designed specifically for these types of interpretation, such as PolyPhen-2 [15]. Because of the importance of understanding which variants are disease-related, programmes such as SNPeffect [16], PhD-SNP [17], PMUT [18], SIFT [19], SNAP [20] [21], SNPs&GO [13] and nsSNPAnalyzer [22] were utilised to predict whether a given single-point protein mutation affected the protein function. For the structural analysis, theoretical models of 124 SNPs of SOD1 were created by comparative modeling using the MHOLline workflow [23], which includes Modeller [24] and Procheck [25]. Models were aligned with the native protein by the TM-align algorithm [26].

Despite progress in protein structure prediction, comparative modeling remains the only method that can reliably predict the 3D structure of a protein with an accuracy that is comparable to that of a low-resolution experimentally determined structure [27]. A model calculated using a template structure that shares more than 30% sequence identity is indicative of an overall accurate structure [28]. In this work, comparative modeling was performed using templates with high-resolution X-ray structures (< 2Å) and more than 99% sequence identity.

As a result, a human-curated database was developed using the server side include in Java, JMOL, for biologists and clinicians to explore SOD1 nsSNPs and the resulting changes in structure and function. This database is freely available at http://bioinfogroup.com/database/ and will be regularly updated.

## Materials and Methods

### Sequence retrieval

The sequence of the human SOD1 [UNIPROT: P00441] were retrieved from the UNIPROT database [29].

### Achievement of the mutant natural variations of SOD1

The 124 natural variants of the human SOD1 that are associated with ALS were obtained from the OMIM database [30], UNIPROT database [29], and ALSOD [31], which provide systematic and in-depth qualitative and quantitative overviews of genetic research in both familial and sporadic ALS.

### Functional analysis of the mutant sequences

The mutant proteins were functionally analyzed using eleven different algorithms: SIFT, nsSNPAnalyzer, SNPs&GO, Polyphen 2, PMUT, PhD-SNP, SNAP, TANGO, WATLZ, LIMBO, and FoldX (Table S1).

### Structural conformation and conservation analysis of SOD1

ConSurf (ConSurf v3.0), a bioinformatics tool based on the phylogenetic relations between homologous sequences, was used to evaluate the conservation at each amino acid position of the SOD1 protein [32,33]. This algorithm included a multiple alignment, in which a minimum BLAST score of 80% homology between the possible options of sequences and the human SOD1 was used as selection criterion for homologous sequences. 

### Comparative modelling

The mutant models were built using the MHOLline workflow [23] with the crystallographic structure of human SOD1 as the template. The TM-scores and root mean square deviations (RMSDs) of the mutant structures with respect to the wild-type structure were calculated using TM-Align [26].

### Creation of the SOD1 database

A human-curated database was developed using the server side include in Java, JMOL. The database at http://bioinfogroup.com/database contains the results obtained from this work. The data contained on the site were individually and manually obtained in each algorithm. 

## Results and Discussion

The motivation for choosing SOD1 is the intimate relation of its variants with the development of ALS and the increasing number of studies that have been performed for greater understanding of this disease and of SNP predictive algorithms. 

In this work, 124 natural variants of SOD1 obtained through three different databases were analyzed. Until now, no single database contained the complete set of *in silico* analyses per mutation, revealing the lack of a more updated and complete database about SOD1. The SOD1 database, http://bioinfogroup.com/database, was created to supplement these demands and form a database of the results found during the execution of this project and from others in the future, allowing researchers and clinicians to explore SOD1 and its variants. Our database, which contains all the currently described mutations, allows the user to evaluate the effects of SOD1 on ALS in a more embracing manner than that of the other existing databases. In addition, the user can know and analyze the algorithms that are most frequently used to predict SNPs, starting from the access to the individual results of the application for each of the 124 existing mutations. With its interactive interface, the SOD1 database allows dynamic utilization by enabling users to select only the results of the mutations and algorithms that are most important to them.

### Functional analysis of the mutant sequences

**Figure 1:**
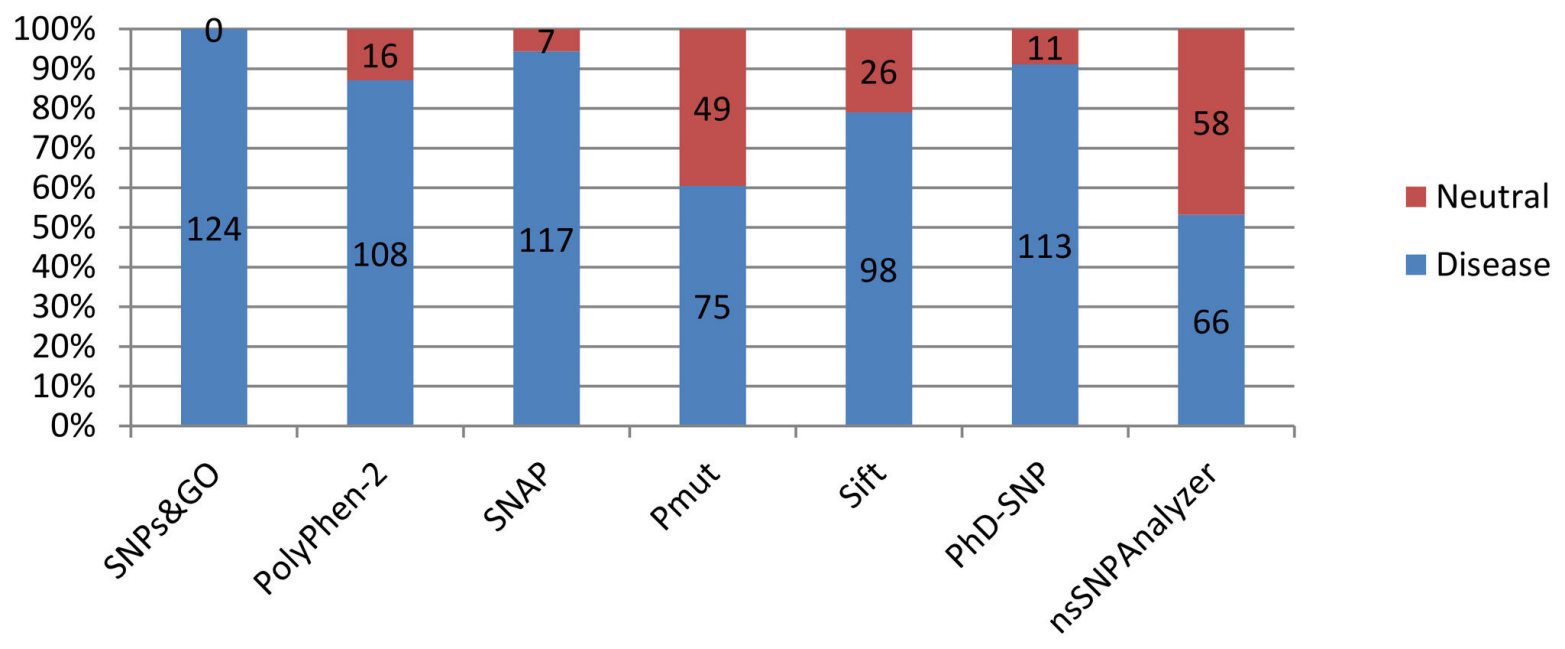
Mutations designated as neutral and non-neutral by the algorithm. Blue bars show the total number of mutations classified as responsible for the disease, while the red bars show the total number of mutations considered neutral.

The information derived from the application of algorithms that predict the pathogenicity of mutations is quantitatively represented in Figure 1 and detailed in Table S2.

SIFT: This method takes a query sequence and uses multiple alignment information to predict tolerated and deleterious substitutions for every position of the query sequence. This is a multistep procedure that, given a protein sequence, (1) searches for similar sequences, (2) chooses closely related sequences that may share similar function, (3) obtains the multiple alignment of these chosen sequences, and (4) calculates normalized probabilities for all possible substitutions at each position from the alignment [19]. Seventy-nine percent of the analyzed mutations, totalizing 98 mutations, were classified as responsible for affecting the proteic function; the others were tolerable. 

nsSNPAnalyzer: A web-based software, nsSNPAnalyzer extracts the structural and evolutionary information from a query nsSNP and uses a machine learning method known as random forest to predict the phenotypic effect of the nsSNP [22]. Nearly fifty-three percent of the analyzed mutations, 66 mutations, were classified as disease originators. The mutations C111Y and E133V obtained unknown results, and the others were classified as neutral.

SNPs&GO: A SVM-based method that, starting from the protein sequence, uses different pieces of information, including that derived from the GO annotation of the protein, to predict whether a given mutation can be classified disease related [13]. One hundred percent of the analyzed mutations were classified as disease originators. 

PolyPhen-2: This method uses eight sequence-based and three structure-based predictive features that were selected automatically by an iterative greedy algorithm. The majority of these features involve a comparison between a property of the wild-type allele and the corresponding property of the mutant allele. The functional importance of an allele replacement is predicted from its individual features by a naive Bayes classifier [15]. Approximately eighty-seven percent of the analyzed mutations, 108 mutations, were classified as possible originators of injury, and the remaining were benign. 

PMUT: This method allows the prediction of the pathological character of single-point amino acid mutation based on the use of neural networks. This method also allows the fast scanning of mutational hot spots, which are obtained using three procedures: (1) alanine scanning, (2) massive mutation and (3) genetically accessible mutations. A graphical interface for Protein Data Bank (PDB) structures, when available, and a database containing hot spot profiles for all non-redundant PDB structures are also accessible from the PMUT server [34]. Nearly sixty percent of the analyzed mutations, 75 mutations, were classified as pathological, and the remaining were neutral.

PhD-SNP: This method is based on support vector machines (SVMs) that starts from the protein sequence information and can predict whether a new phenotype derived from a nsSNP can be related to a genetic disease in humans, using a dataset of 21185 single point mutations [17]. Nearly ninety-one percent of the analyzed mutations (113 mutations) were classified as disease originators, and the remaining were neutral. 

SNAP: This method utilizes various biophysical characteristics of the substitution, as well as evolutionary information, some predicted (or observed, when available) structural features, and possibly annotations, to predict whether a mutation is likely to alter protein function (in either direction: gain or loss) [20]. Nearly ninety-four percent of the analyzed mutations (117 mutations) were classified as non-neutral, and the remaining were neutral. 

SNPeffect: This method uses sequence- and structure-based bioinformatics tools to predict the effect of protein-coding SNVs on the structural phenotype of proteins. SNPeffect integrates aggregation prediction (TANGO) [35], amyloid prediction (WALTZ) [36], chaperone-binding prediction (LIMBO) [37] and protein stability analysis (FoldX) [38] for structural phenotyping [16]:

TANGO: Six mutations were classified as causing an increased tendency for protein aggregation, and 5 were classified as a decrease of that tendency. The other 115 mutations were classified as not changing the protein aggregation from that of the wild-type SOD1.

WALTZ: Only two mutations were classified as responsible for increasing the amyloid propensity and two for its decrease. The remaining approximately ninety-seven percent was classified as having no changes concerning this parameter.

LIMBO: None of the mutations were classified as causing changes in chaperone binding compared with the wild-type SOD1.

FoldX: Approximately eighty-six percent of the mutations were classified as responsible for the decrease of protein stability, 4 mutations as causing an increase thereof, and only 13 mutations were found to be unaffected compared with the non-mutated SOD1.

Three histidine residues (HIS46, HIS48, HIS80) and one glycine residue (G85R) that formed the ligation site between metal and SOD1 were affected by ALS mutations. All of these mutations were considered pathological according to the seven algorithms for predicting pathogenicity. Furthermore, these mutant proteins did not exhibit the tendency to either aggregate or form amyloids and did not alter chaperone binding. However, their protein stability was considerably reduced in comparison to that of the wild-type SOD1. These results are not consistent with those of other experimental studies, which have shown that H46R and G85R are among the most stable ALS-related mutations [39].

**Figure 2:**
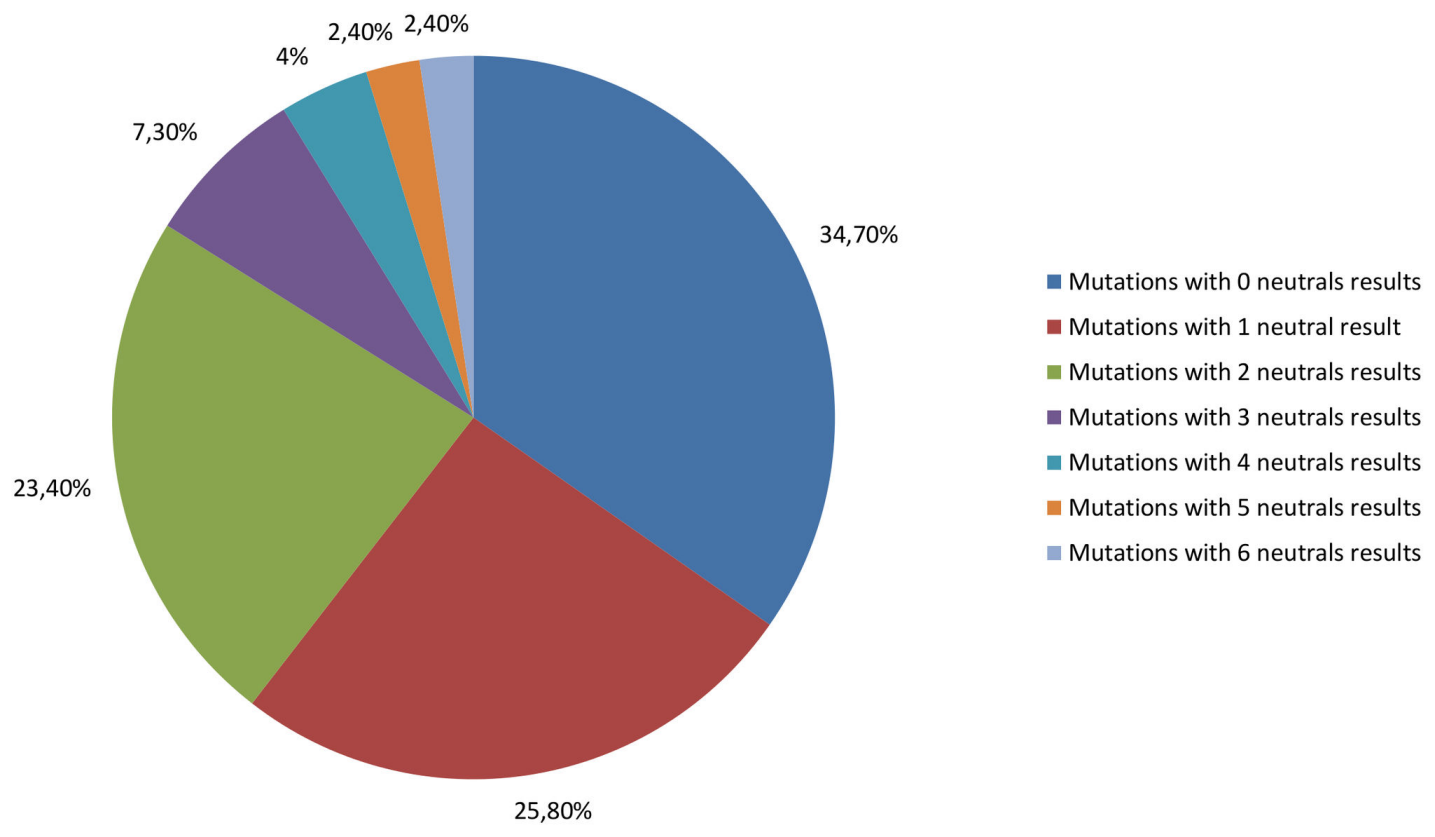
Percentage of mutations with neutral results obtained by the algorithms used for analysis of SOD1. Blue bar represents the percentage of mutations that, among the seven used algorithms, presented no neutral result and were therefore considered malign by all algorithms. Red bar, the percentage of mutations with neutral results; Green bar, 2 neutral results; Purple bar, 3 neutral results; Yellow bar, 5 neutral results; Orange bar, 6 neutral results; Brown bar, 7 neutral results.

Starting from the SNP analysis from using the seven algorithms individually, only 43 natural variants (34.7%) were classified as pathological by all of the algorithms. Of the remaining variants, 25.8% were classified as neutral, 23.4% had two classifications, and the other 16.1% had between three and six (Figure 2). No mutations were classified as neutral by all algorithms, and SNPs&GO was the only algorithm that classified all the variants as malignant, and therefore the one which presented higher accuracy in the classifications of the mutations that cause amyotrophic lateral sclerosis. In contrast, the nsSNPAnalyzer classified almost half of the mutations (46.7%) as benign, showing less accuracy in the identification of the SNPs that cause ALS. The mutations were also analyzed at the same time by all algorithms. Considering the number of benign and malign results for each mutation, the total hit tax on the prediction of the 124 mutations was approximately 91%. The classification differences observed by the mutations occur because each algorithm has different parameters of prediction, which illustrates the requirement of using more than one algorithm to obtain greater reliability in the prediction of non-described SNPs.

Interestingly, all the mutations that occurred on the binding site to metal of the SOD1 were considered pathological by the 7 algorithms that were used. Therefore, we can hypothesize that these mutations have the functional localization on the protein as a common parameter, which is very reasonable considering that cellular functions are performed by 3D well-folded protein structures and protein-protein and protein-ligand interactions [40] and that alterations of these parameters may cause a loss or alteration of proteic function. Moreover, in a manner consistent with experimental results, the A4V variant, which was described as responsible by the most severe form of fALS [41], was considered pathological by all the algorithms except for the nsSNPAnalyzer.

Regarding the analysis performed by SNPeffect, the vast majority of the mutations were classified as having no significant changes in relation to the protein aggregation tendency, amyloid propensity tendency or changes in the binding of chaperones. Conversely, according to the protein stability analysis parameter, most of the mutations (86.3%) were classified as being less stable than the wild-type SOD1. These results are extremely important in understanding the probable pathological change whereby mutations lead to the development of ALS. In particular, this analysis indicated that the mutation A4V exhibited not only a reduced protein stability but also an increased tendency to aggregate, corroborating experimental data that highlighted the greater severity of this mutation over those of other mutations, consistent with experimental results indicating that the mutant A4V more easily aggregates in the presence of cupric ions under copper-mediated oxidative conditions than does wild-type SOD1 [42]. In contrast, the mutant G93A, which presents an initial rate of oligomerization greater than twice that of wild-type SOD1 during experimental analysis [43], was classified by SNPeffect as being unaffected by protein aggregation. 

### Structural conformation and conservation analysis of SOD1

**Figure 3:**
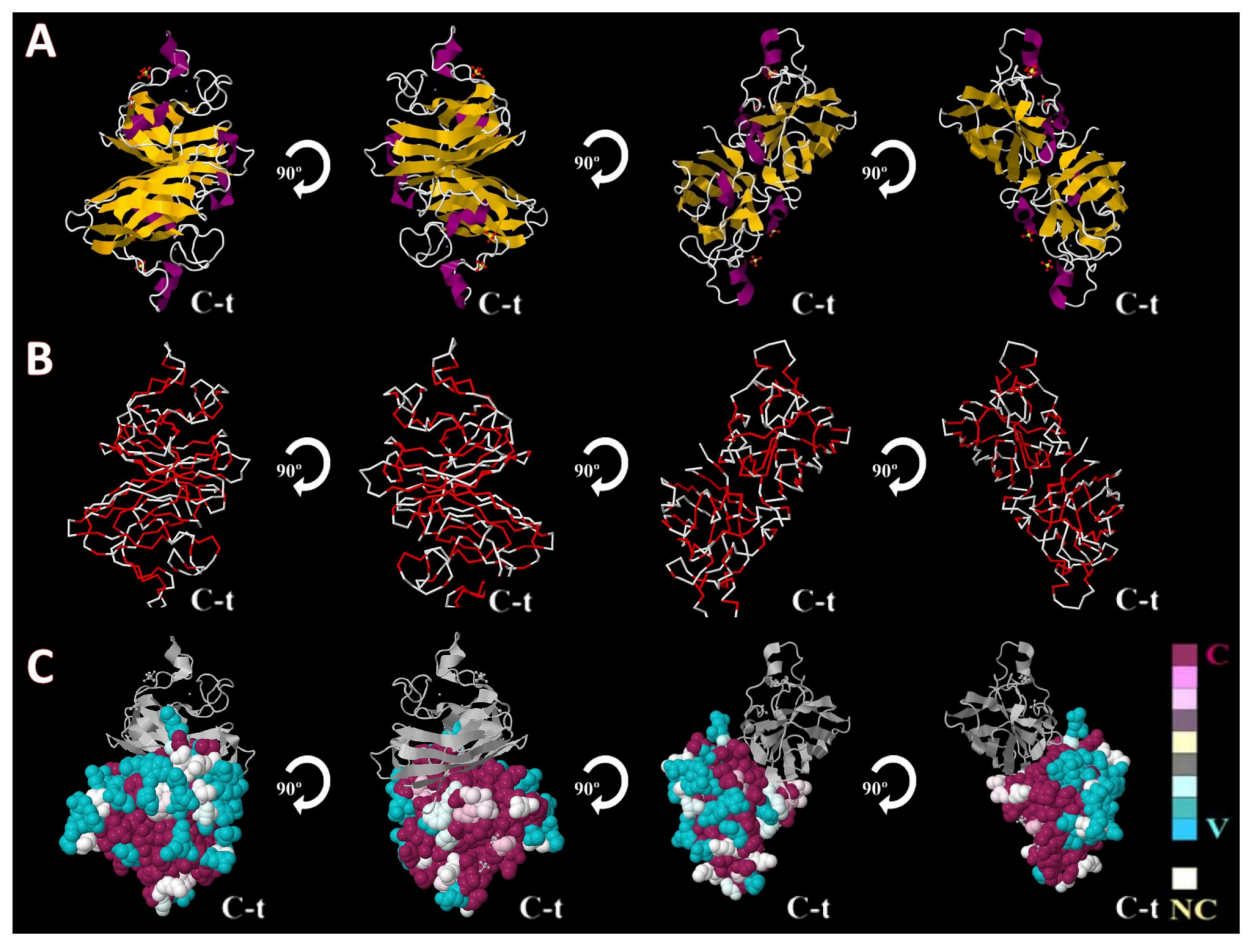
Structural conformation and conservational analysis of the human SOD1. (A) Structural representation of the three-dimensional model of the SOD1 [PDB: 2VOA] in a form of a diagram in cartoon. Different elements of the secondary structure are in various colors: in magenta, the alpha helix; in yellow arrows, beta sheets; and in white lines, coils. (B) Three-dimensional structure of human SOD1, represented in backbone form of, with mutations described on the literature marked in red. (C) Conservation profile of human SOD1 using ConSurf conservation analysis. The protein was visualized using Jmol with color-coded conservations. The conserved and variable residues are presented as space-filled models and colored according to the conservation scores. Each structure is turned 90 degrees to show the different sides of the protein.

**Figure 4:**
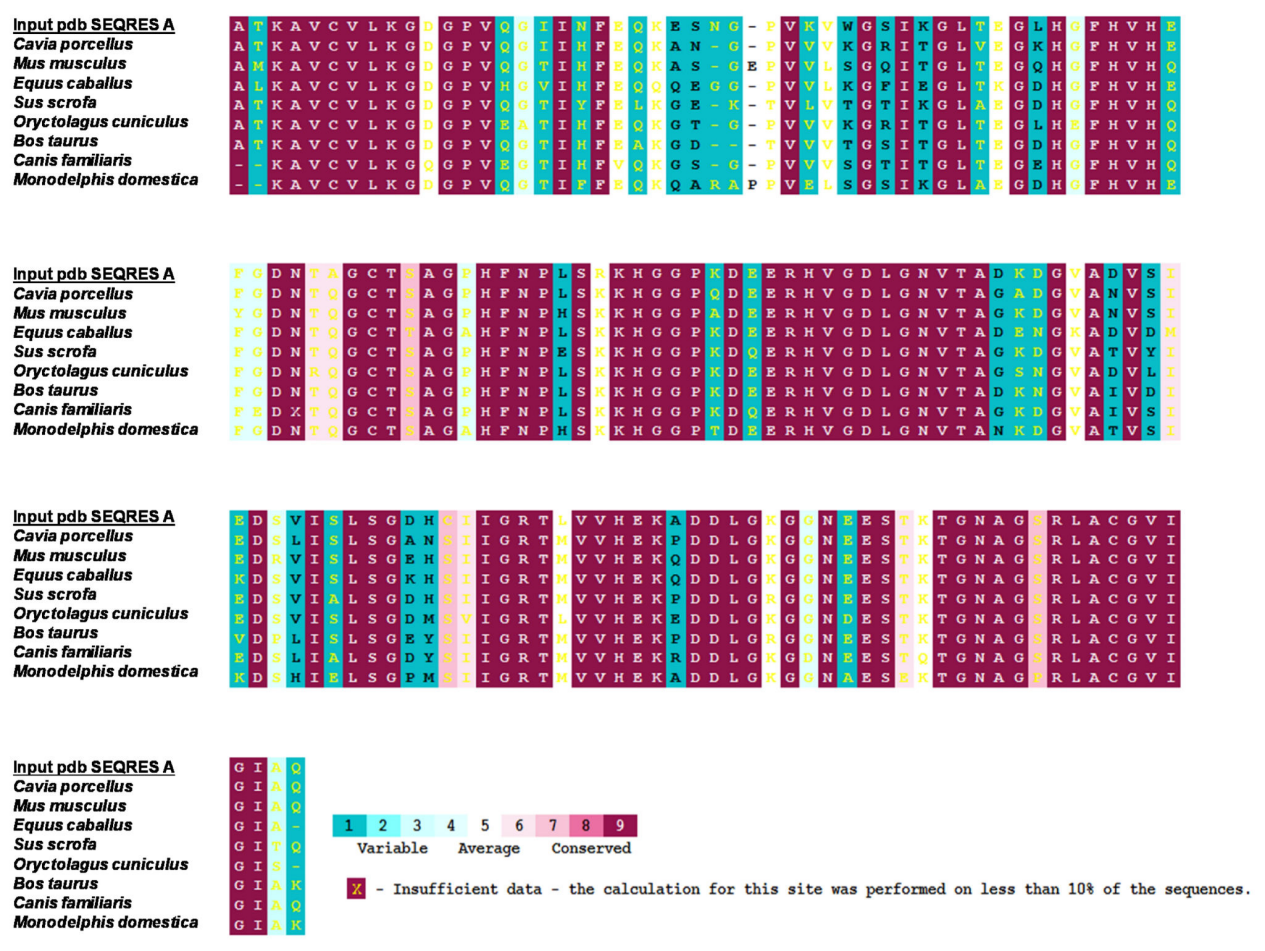
Multiple Sequence Alignment Color-Coded by Conservation. The ConSurf algorithm was used to provide conservation score for the amino acids of superoxide dismutase 1, starting from the multiple alignment of sequences of SOD1 from 8 different mammalian species that had between 80 and 100% homology with the human SOD1. The color-coding bar shows the coloring scheme: conserved amino acids are colored bordeaux, residues of average conservation are white, and variable amino acids are turquoise. Two columns are basically formed by gaps: at the position of amino acid 26, the gaps are because of sequences of human SOD1, *Equus caballus* and *Morrodelphis domestica*; while at amino acid position 28, the gaps are because of the species *Mus musculus* and *Morrodelphis domestica*. In addition, it is verified that 61.43% of the amino acids are highly conserved between the analyzed species. The asterisks on the superior region of the alignment delimit the amino acids that have mutations described in the literature.

The results generated by the ConSurf tool consist of a structural representation of the protein (Figure 3) and a multiple alignment of the sequences (Figure 4). Both contain a colorimetric conservation score, in which conserved amino acids are colored bordeaux, residues of average conservation are white, and variable amino acids are turquoise. It was verified that 61.4% of the amino acids from SOD1 were totally conserved, including all amino acids that formed the bonding site to metal (HIS46, HIS48, HIS63, HIS71, HIS80, ASP83 and HIS120).

Evolutionary information is of fundamental importance for detecting mutations that affect human health [44]. ConSurf identifies functional regions in proteins, taking into account the evolutionary relationships among their sequence homologues [32]. The ConSurf conservation analysis was performed by evolutionarily related conservation scores of the residues for functional region identification from proteins of known three-dimensional structures [40]. For the identification of functional regions for mapping the phylogenetic information between the homologues of SOD1, we formulated a BLAST [45] with the criterion of selecting only sequences with homology between 80 and 100% for the alignment. This criterion allowed us to select only sequences that were evolutionally close to the human SOD1 protein. Consequently, the alignment was performed only in mammalian enzymes. The results verified that most of the amino acids, 61.4%, were highly conserved between the analyzed species. The ConSurf analysis also revealed, as expected, that the functional regions of the protein are highly conserved; it classified all the amino acids of the binding site to metal on SOD1, including the three histidines that have mutations related to the development of ALS, with the highest possible score of conservation (score = 9). In addition, (Figure 4) two columns became aligned by gaps at the positions of amino acids 26 and 28. The first gap is a result of the sequences of the human SOD1, *Equus caballus* and *Morrodelphis domestica*; while the second is because of the *Mus musculus* and *Morrodelphis domestica* species. 

When comparing the results obtained from the SNP and ConSurf analysis algorithms, all mutations that were classified as benign (Figure 2) by at least 5 algorithms (D96N, I99V, L117V e N19S, D96V, E100K) occurred in amino acids considered non-conserved by ConSurf. 

### Comparative modelling

The natural variants were substituted into the wild-type sequence for comparative modelling. These sequences were submitted to the MHOLline workflow [23]. An alignment between the native and mutant structures was performed using TM-Align [26]. Parameters such as the TM-score and root mean square deviation (RMSD) were used to analyse the topology and structural similarity of the models. TM-score was used to assess the topological similarity of two protein structures, while RMSD was the measure of the average distance between the backbones of the superimposed proteins [40]. The RMSD values for the modelled mutants were significant for pathogenicity for all missense mutations (http://bioinfogroup.com/database). RMSD values greater than 0.15 were considered significant structural perturbations that could have functional implications for the protein [46].

### SOD1 database

**Figure 5:**
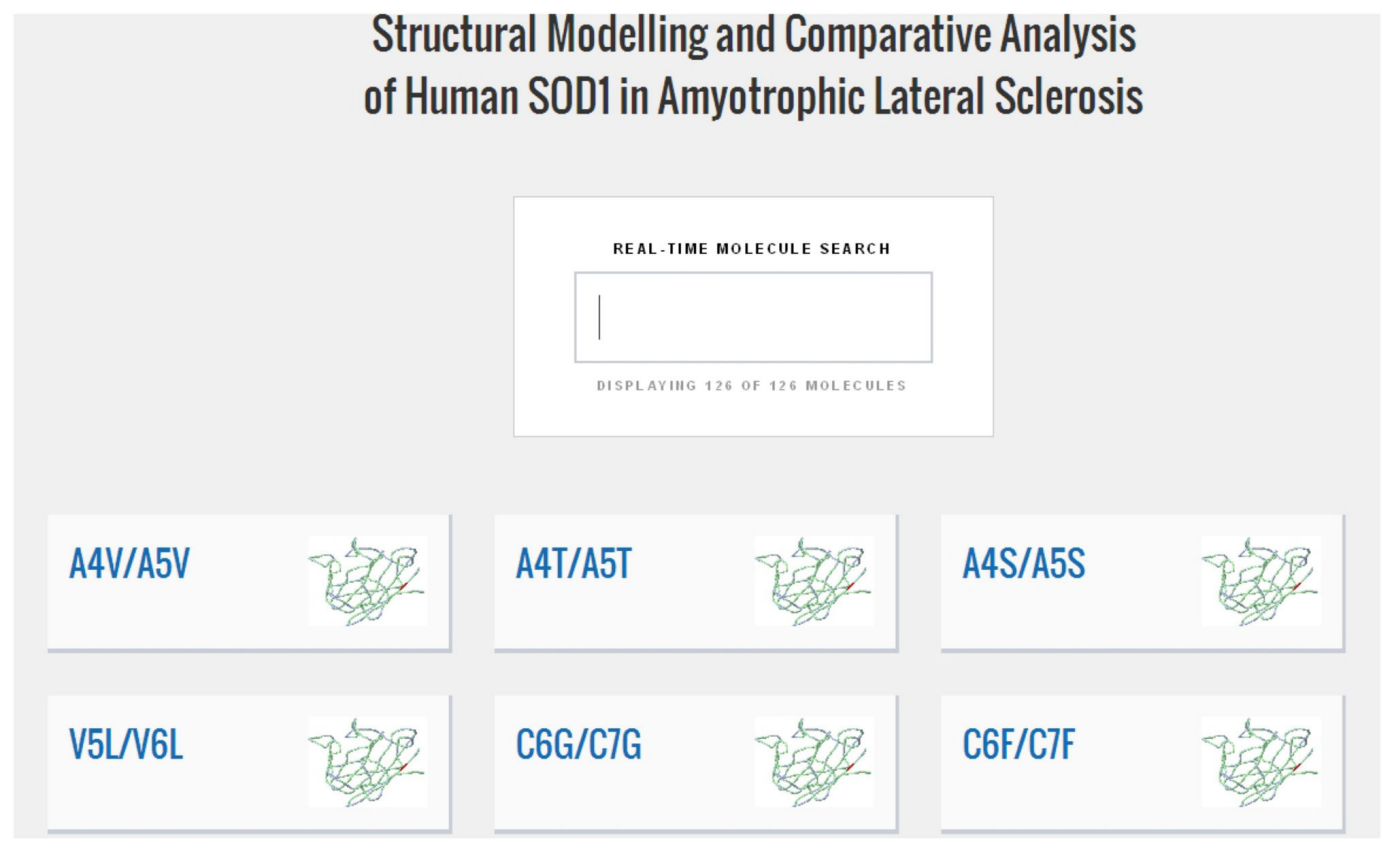
Screenshot of the SOD1 Database web interface for structural modelling and comparative analysis.

All results obtained through this work are available for search on our free SOD1 database at http://bioinfogroup.com/database. The database interface (Figure 5) allows users to search for a mutation by its non-synonymous SNP. The SOD1 database allows a user to quickly retrieve and rapidly analyse the predicted effects of protein variants. In addition to predicting the effects of variants, an alignment of the wild-type and mutant structures can be visualised using the database.

## Conclusions

In general, the accuracies of the prediction tools (SNAP, SNPs&GO, SIFT, PolyPhen-2, nsSNPAnalyzer, PMUT, PhD-SNP) are estimated to be between 50% and 80% [20,47]. In this work, we obtained an extra total average hit tax between the algorithms of 80.8%. This significant value that shows that to obtain greater reliability, it is necessary to use more than one tool to predict SNPs. In the case of SOD1, only 34.7% of the mutations presented pathogenicity with all of the tested algorithms. The algorithm with more accuracy was SNPs&GO, which uses information derived from the GO annotation of the protein to predict whether a given mutation can be classified disease-related [13]. When applying seven distinct algorithms to predict 124 SNPs, the results indicate that the majority of natural mutants are classified as harmful to protein structure and thus corroborate the correlation between the mutations and fALS. We also observed that the results of the algorithms diverged, although all were based on similar calculation mechanisms. Moreover, the results found by the pathogenicity prediction tools are consistent with those found when analyzing the conservation of the amino acids. The analysis performed using the SNPeffect algorithm allows us to infer that the main protein parameter responsible for causing ALS might be the instability of the mutated SOD1. This inference stems from the fact that according to the protein stability analysis parameter, most of the mutations (86.3%) were classified as being less stable than the wild-type SOD1. Furthermore, we can also conclude that chaperone binding is not related to the development of ALS because none of the mutations was classified as causing changes in chaperone binding compared with the wild-type SOD1.

The resulting database allows biologists and clinicians to explore SOD1 nsSNPs and their functional inferences. It is a vast resource for the molecular analysis of amyotrophic lateral sclerosis, which allows a user to expand his knowledge of the molecular basis of the disease.

## Supporting Information

Table S1 **Tools for *In Silico* Analysis of Missense Substitutions.**
(PDF)

Table S2 **Functional Analysis of SOD1 in fALS.**
(PDF)

## References

[1] Andersen PM (2006) Amyotrophic lateral sclerosis associated with mutations in the CuZn superoxide dismutase gene. Curr Neurol Neurosci Rep 6: 37-46. doi:10.1007/s11910-996-0008-9. PubMed: 16469270. 16469270

[2] Phukan J, Hardiman O (2009) The management of amyotrophic lateral sclerosis. J Neurol 256: 176-186. doi:10.1007/s00415-009-0142-9. PubMed: 19224316. 19224316

[3] Garbuzova-Davis S, Rodrigues MC, Hernandez-Ontiveros DG, Louis MK, Willing AE et al. (2011) Amyotrophic lateral sclerosis: a neurovascular disease. Brain Res 1398: 113-125. doi:10.1016/j.brainres.2011.04.049. PubMed: 21632035. 21632035

[4] Valentine JS, Doucette PA, Zittin Potter S (2005) Copper-zinc superoxide dismutase and amyotrophic lateral sclerosis. Annu Rev Biochem 74: 563-593. doi:10.1146/annurev.biochem.72.121801.161647. PubMed: 15952898. 15952898

[5] Chiti F, Dobson CM (2006) Protein misfolding, functional amyloid, and human disease. Annu Rev Biochem 75: 333-366. doi:10.1146/annurev.biochem.75.101304.123901. PubMed: 16756495. 16756495

[6] Nordlund A, Leinartaite L, Saraboji K, Aisenbrey C, Gröbner G et al. (2009) Functional features cause misfolding of the ALS-provoking enzyme SOD1. Proc Natl Acad Sci U S A 106: 9667-9672. doi:10.1073/pnas.0812046106. PubMed: 19497878. 19497878 PMC2701049

[7] Lindberg MJ, Byström R, Boknäs N, Andersen PM, Oliveberg M (2005) Systematically perturbed folding patterns of amyotrophic lateral sclerosis (ALS)-associated SOD1 mutants. Proc Natl Acad Sci U S A 102: 9754-9759. doi:10.1073/pnas.0501957102. PubMed: 15987780. 15987780 PMC1174986

[8] Shaw BF, Valentine JS (2007) How do ALS-associated mutations in superoxide dismutase 1 promote aggregation of the protein? Trends Biochem Sci 32: 78-85. doi:10.1016/j.tibs.2006.12.005. PubMed: 17208444. 17208444

[9] Cudkowicz ME, McKenna-Yasek D, Sapp PE, Chin W, Geller B et al. (1997) Epidemiology of mutations in superoxide dismutase in amyotrophic lateral sclerosis. Ann Neurol 41: 210-221. doi:10.1002/ana.410410212. PubMed: 9029070. 9029070

[10] Deng HX, Hentati A, Tainer JA, Iqbal Z, Cayabyab A et al. (1993) Amyotrophic lateral sclerosis and structural defects in Cu,Zn superoxide dismutase. Science 261: 1047-1051. doi:10.1126/science.8351519. PubMed: 8351519. 8351519

[11] Rodriguez JA, Valentine JS, Eggers DK, Roe JA, Tiwari A et al. (2002) Familial amyotrophic lateral sclerosis-associated mutations decrease the thermal stability of distinctly metallated species of human copper/zinc superoxide dismutase. J Biol Chem 277: 15932-15937. doi:10.1074/jbc.M112088200. PubMed: 11854285. 11854285

[12] Alkan C, Coe BP, Eichler EE (2011) Genome structural variation discovery and genotyping. Nat Rev Genet 12: 363-376. doi:10.1038/nrg2958. PubMed: 21358748. 21358748 PMC4108431

[13] Calabrese R, Capriotti E, Fariselli P, Martelli PL, Casadio R (2009) Functional annotations improve the predictive score of human disease-related mutations in proteins. Hum Mutat 30: 1237-1244. doi:10.1002/humu.21047. PubMed: 19514061. 19514061

[14] Carvalho MDC, De Mesquita JF (2013) Structural Modelling and In Silico Analysis of Human Superoxide Dismutase 2. PLOS ONE 8: e65558. doi:10.1371/journal.pone.0065558. PubMed: 23785434. 23785434 PMC3681941

[15] Adzhubei IA, Schmidt S, Peshkin L, Ramensky VE, Gerasimova A et al. (2010) A method and server for predicting damaging missense mutations. Nat Methods 7: 248-249. doi:10.1038/nmeth0410-248. PubMed: 20354512. 20354512 PMC2855889

[16] De Baets G, Van Durme J, Reumers J, Maurer-Stroh S, Vanhee P, et al. (2012) SNPeffect 4.0: on-line prediction of molecular and structural effects of protein-coding variants. Nucleic Acids Res 40: D935-939. 22075996 10.1093/nar/gkr996 PMC3245173

[17] Capriotti E, Calabrese R, Casadio R (2006) Predicting the insurgence of human genetic diseases associated to single point protein mutations with support vector machines and evolutionary information. Bioinformatics 22: 2729-2734. doi:10.1093/bioinformatics/btl423. PubMed: 16895930. 16895930

[18] Ferrer-Costa C, Orozco M, de la Cruz X (2002) Characterization of disease-associated single amino acid polymorphisms in terms of sequence and structure properties. J Mol Biol 315: 771-786. doi:10.1006/jmbi.2001.5255. PubMed: 11812146. 11812146

[19] Ng PC, Henikoff S (2001) Predicting deleterious amino acid substitutions. Genome Res 11: 863-874. doi:10.1101/gr.176601. PubMed: 11337480. 11337480 PMC311071

[20] Bromberg Y, Rost B (2007) SNAP: predict effect of non-synonymous polymorphisms on function. Nucleic Acids Res 35: 3823-3835. doi:10.1093/nar/gkm238. PubMed: 17526529. 17526529 PMC1920242

[21] Bromberg Y, Yachdav G, Rost B (2008) SNAP predicts effect of mutations on protein function. Bioinformatics 24: 2397-2398. doi:10.1093/bioinformatics/btn435. PubMed: 18757876. 18757876 PMC2562009

[22] Bao L, Zhou M, Cui Y (2005) nsSNPAnalyzer: identifying disease-associated nonsynonymous single nucleotide polymorphisms. Nucleic Acids Res 33: W480-W482. doi:10.1093/nar/gki372. PubMed: 15980516. 15980516 PMC1160133

[23] Capriles PV, Guimarães AC, Otto TD, Miranda AB, Dardenne LE et al. (2010) Structural modelling and comparative analysis of homologous, analogous and specific proteins from Trypanosoma cruzi versus Homo sapiens: putative drug targets for chagas' disease treatment. BMC Genomics 11: 610. doi:10.1186/1471-2164-11-610. PubMed: 21034488. 21034488 PMC3091751

[24] Sánchez R, Sali A (1997) Evaluation of comparative protein structure modeling by MODELLER-3. Proteins Suppl 1: 50-58. PubMed: 9485495. 10.1002/(sici)1097-0134(1997)1+<50::aid-prot8>3.3.co;2-w 9485495

[25] Laskowski RA, MacArthur MW, Moss DS, Thornton JM (1993) PROCHECK: a program to check the stereochemical quality of protein structures. Journal of Applied Crystallography 26: 283-291. doi:10.1107/S0021889892009944.

[26] Zhang Y, Skolnick J (2005) TM-align: a protein structure alignment algorithm based on the TM-score. Nucleic Acids Res 33: 2302-2309. doi:10.1093/nar/gki524. PubMed: 15849316. 15849316 PMC1084323

[27] Fiser A, Sali A (2003) Modeller: generation and refinement of homology-based protein structure models. Methods Enzymol 374: 461-491. doi:10.1016/S0076-6879(03)74020-8. PubMed: 14696385. 14696385

[28] Eswar N, Webb B, Marti-Renom MA, Madhusudhan MS, Eramian D, et al. (2007) Comparative protein structure modeling using MODELLER. Curr Protoc Protein Sci Chapter 2: Unit 2 9 10.1002/0471140864.ps0209s50 18429317

[29] UniProt (2013) Update on activities at the Universal Protein Resource (UniProt) in 2013. Nucleic Acids Res 41: D43-D47. doi:10.1093/nar/gks902. PubMed: 23161681. 23161681 PMC3531094

[30] Amberger J, Bocchini C, Hamosh A (2011) A new face and new challenges for Online Mendelian Inheritance in Man (OMIM(R)). Hum Mutat 32: 564-567. doi:10.1002/humu.21466. PubMed: 21472891. 21472891

[31] Lill CM, Abel O, Bertram L, Al-Chalabi A (2011) Keeping up with genetic discoveries in amyotrophic lateral sclerosis: the ALSoD and ALSGene databases. Amyotroph Lateral Scler 12: 238-249. doi:10.3109/17482968.2011.584629. PubMed: 21702733. 21702733

[32] Glaser F, Pupko T, Paz I, Bell RE, Bechor-Shental D et al. (2003) ConSurf: identification of functional regions in proteins by surface-mapping of phylogenetic information. Bioinformatics 19: 163-164. doi:10.1093/bioinformatics/19.1.163. PubMed: 12499312. 12499312

[33] Ashkenazy H, Erez E, Martz E, Pupko T, Ben-Tal N (2010) ConSurf 2010: calculating evolutionary conservation in sequence and structure of proteins and nucleic acids. Nucleic Acids Res 38: W529-W533. doi:10.1093/nar/gkq399. PubMed: 20478830. 20478830 PMC2896094

[34] Ferrer-Costa C, Gelpí JL, Zamakola L, Parraga I, de la Cruz X et al. (2005) PMUT: a web-based tool for the annotation of pathological mutations on proteins. Bioinformatics 21: 3176-3178. doi:10.1093/bioinformatics/bti486. PubMed: 15879453. 15879453

[35] Fernandez-Escamilla AM, Rousseau F, Schymkowitz J, Serrano L (2004) Prediction of sequence-dependent and mutational effects on the aggregation of peptides and proteins. Nat Biotechnol 22: 1302-1306. doi:10.1038/nbt1012. PubMed: 15361882. 15361882

[36] Maurer-Stroh S, Debulpaep M, Kuemmerer N, Lopez de la Paz M, Martins IC et al. (2010) Exploring the sequence determinants of amyloid structure using position-specific scoring matrices. Nat Methods 7: 237-242. doi:10.1038/nmeth.1432. PubMed: 20154676. 20154676

[37] Van Durme J, Maurer-Stroh S, Gallardo R, Wilkinson H, Rousseau F et al. (2009) Accurate prediction of DnaK-peptide binding via homology modelling and experimental data. PLoS Comput Biol 5: e1000475 PubMed: 19696878. 19696878 10.1371/journal.pcbi.1000475 PMC2717214

[38] Schymkowitz J, Borg J, Stricher F, Nys R, Rousseau F et al. (2005) The FoldX web server: an online force field. Nucleic Acids Res 33: W382-W388. doi:10.1093/nar/gki387. PubMed: 15980494. 15980494 PMC1160148

[39] Vassall KA, Stubbs HR, Primmer HA, Tong MS, Sullivan SM et al. (2011) Decreased stability and increased formation of soluble aggregates by immature superoxide dismutase do not account for disease severity in ALS. Proc Natl Acad Sci U S A 108: 2210-2215. doi:10.1073/pnas.0913021108. PubMed: 21257910. 21257910 PMC3038722

[40] Jimenez-Lopez JC, Gachomo EW, Seufferheld MJ, Kotchoni SO (2010) The maize ALDH protein superfamily: linking structural features to functional specificities. BMC Struct Biol 10: 43. doi:10.1186/1472-6807-10-43. PubMed: 21190582. 21190582 PMC3022562

[41] Siddique N, Siddique T (2008) Genetics of amyotrophic lateral sclerosis. Phys Med Rehabil Clin N Am 19: 429-439, vii 18625408 10.1016/j.pmr.2008.05.001 PMC2553626

[42] Li C, Xu WC, Xie ZS, Pan K, Hu J et al. (2013) Cupric ions induce the oxidation and trigger the aggregation of human superoxide dismutase 1. PLOS ONE 8: e65287. doi:10.1371/journal.pone.0065287. PubMed: 23755211. 23755211 PMC3670862

[43] Banci L, Bertini I, Boca M, Girotto S, Martinelli M et al. (2008) SOD1 and amyotrophic lateral sclerosis: mutations and oligomerization. PLOS ONE 3: e1677. doi:10.1371/journal.pone.0001677. PubMed: 18301754. 18301754 PMC2250751

[44] Ramensky V, Bork P, Sunyaev S (2002) Human non-synonymous SNPs: server and survey. Nucleic Acids Res 30: 3894-3900. doi:10.1093/nar/gkf493. PubMed: 12202775. 12202775 PMC137415

[45] Altschul SF, Wootton JC, Gertz EM, Agarwala R, Morgulis A et al. (2005) Protein database searches using compositionally adjusted substitution matrices. FEBS J 272: 5101-5109. doi:10.1111/j.1742-4658.2005.04945.x. PubMed: 16218944. 16218944 PMC1343503

[46] Mistri M, Tamhankar PM, Sheth F, Sanghavi D, Kondurkar P et al. (2012) Identification of novel mutations in HEXA gene in children affected with Tay Sachs disease from India. PLOS ONE 7: e39122. doi:10.1371/journal.pone.0039122. PubMed: 22723944. 22723944 PMC3377590

[47] Ng PC, Henikoff S (2006) Predicting the effects of amino acid substitutions on protein function. Annu Rev Genomics Hum Genet 7: 61-80. doi:10.1146/annurev.genom.7.080505.115630. PubMed: 16824020. 16824020

