# Supplementary material for: Structural and Functional Analysis of Human SOD1 in Amyotrophic Lateral Sclerosis
Source: PLoS One. 2013 Dec 2;8(12):e81979. doi: 10.1371/journal.pone.0081979 (PMC3846731; doi:10.1371/journal.pone.0081979)
Supplement: Table S1 — Tools for In Silico Analysis of Missense Substitutions. (PDF) [file pone.0081979.s001.pdf]

Tools for *In Silico* Analysis of Missense Substitutions

Program Name	Function Principle	URL	Key references
SNPs&GO	An accurate method that, starting from a protein sequence, can predict whether a mutation is disease related or not by exploiting the protein functional annotation.	http://snps-and-go.biocomp.unibo.it/snps-and-go/	(Calabrese, Capriotti et al. 2009)
PolyPhen-2	Use for predicting damaging effects of missense mutations, uses eight sequence-based and three structure-based predictive features, which were selected automatically by an iterative greedy algorithm.	http://genetics.bwh.harvard.edu/pph2/	(Adzhubei, Schmidt et al. 2010)
SNAP	A neural network-based method for the prediction of the functional effects of non-synonymous SNPs. SNAP needs only sequence information as input, but benefits from functional and structural annotations, if available.	http://www.rostlab.org/services/SNAP/	(Bromberg and Rost 2007)
Pmut	Allows the fast and accurate prediction (~80% success rate in humans) of the pathological character of single point amino acidic mutations based on the use of neural networks.	http://mmb2.pcb.ub.es:8080/Pmut/	(Ferrer-Costa, Orozco et al. 2002)
SIFT	A tool that uses sequence homology to predict whether a substitution affects protein function, which sorts intolerant from tolerant substitutions, classifies substitutions as tolerated or deleterious.	http://sift.bii.a-star.edu.sg/	(Ng and Henikoff 2001)
PhD-SNP	A method to predict whether a given single point protein mutation is related to a human disease or not.	http://snps.uib.es/phd-snp/phd-snp.html	(Capriotti, Calabrese et al. 2006)

nsSNPAnalyzer	To facilitate identifying disease-associated nsSNPs from a large number of neutral nsSNPs, nsSNPANalyzer is important to develop computational tools to predict the nsSNP's phenotypic effect (disease-associated versus neutral).	http://snpanalyzer.uthsc.edu/	(Bao, Zhou et al. 2005)
ConSurf and ConSeq	Are two well-established web servers for calculating the evolutionary conservation of amino acid positions in proteins using an empirical Bayesian inference, starting from protein structure and sequence, respectively.	http://consurf.tau.ac.il/	(Ashkenazy, Erez et al. 2010)
