# Supplementary material for: Structural and Functional Analysis of Human SOD1 in Amyotrophic Lateral Sclerosis
Source: PLoS One. 2013 Dec 2;8(12):e81979. doi: 10.1371/journal.pone.0081979 (PMC3846731; doi:10.1371/journal.pone.0081979)
Supplement: Table S2 — Functional Analysis of SOD1 in fALS. (PDF) [file pone.0081979.s002.pdf]

Functional Analysis of SOD1 in fALS

Natural variant	SNPs&GO	Polyphen2	SNAP	PMUT	SIFT	PhD-SNP	nsSNP Analyzer	Aggregation Tendency (TANGO)	Amyloid Propensity (WALTZ)	Chaperone Binding Tendency (LIMBO)	Protein Stability (FoldX)
Ala4Val	Disease	Probably damaging	Non neutral	Pathological	Affect protein function	Disease	Neutral	Increases	Not afected	Not afected	Reduces
Ala4Thr	Disease	Probably damaging	Non neutral	Pathological	Affect protein function	Disease	Neutral	Not afected	Not afected	Not afected	Reduces
Ala4Ser	Disease	Benign	Non neutral	Neutral	Affect protein function	Disease	Neutral	Not afected	Not afected	Not afected	Reduces
Val5Leu	Disease	Probably damaging	Neutral	Pathological	Affect protein function	Disease	Neutral	Not afected	Not afected	Not afected	Not afected
Cys6Gly	Disease	Probably damaging	Non neutral	Neutral	Affect protein function	Disease	Disease	Not afected	Not afected	Not afected	Reduces
Cys6Phe	Disease	Probably damaging	Non neutral	Pathological	Affect protein function	Disease	Disease	Increases	Not afected	Not afected	Severely reduces
Cys6Ser	Disease	Probably damaging	Non neutral	Pathological	Affect protein function	Disease	Disease	Not afected	Not afected	Not afected	Reduces
Val7Glu	Disease	Probably damaging	Non neutral	Pathological	Affect protein function	Disease	Neutral	Not afected	Not afected	Not afected	Slightly reduces
Leu8Gln	Disease	Probably damaging	Non neutral	Pathological	Affect protein function	Disease	Disease	Not afected	Not afected	Not afected	Reduces
Leu8Val	Disease	Probably damaging	Non neutral	Neutral	Affect protein function	Disease	Neutral	Not afected	Not afected	Not afected	Reduces
Gly10Val	Disease	Probably damaging	Non neutral	Neutral	Affect protein function	Disease	Disease	Not afected	Not afected	Not afected	Severely reduces
Gly10Arg	Disease	Probably damaging	Non neutral	Pathological	Affect protein function	Disease	Disease	Not afected	Not afected	Not afected	Severely reduces
Gly12Arg	Disease	Probably damaging	Non neutral	Pathological	Tolerated	Disease	Disease	Not afected	Not afected	Not afected	Slightly reduces
Val14Met	Disease	Probably damaging	Non neutral	Neutral	Affect protein function	Disease	Neutral	Not afected	Decreases	Not afected	Reduces

Val14Gly	Disease	Probably damaging	Non neutral	Neutral	Affect protein function	Disease	Neutral	Not afected	Not afected	Not afected	Reduces
Gly16Ala	Disease	Probably damaging	Non neutral	Pathological	Affect protein function	Disease	Disease	Not afected	Increases	Not afected	Severely reduces
Gly16Ser	Disease	Probably damaging	Non neutral	Pathological	Affect protein function	Disease	Disease	Not afected	Increases	Not afected	Severely reduces
Asn19Ser	Disease	Benign	Neutral	Neutral	Tolerated	Neutral	Disease	Not afected	Decreases	Not afected	Reduces
Phe20Cys	Disease	Probably damaging	Non neutral	Pathological	Affect protein function	Disease	Disease	Not afected	Not afected	Not afected	Reduces
Glu21Lys	Disease	Benign	Non neutral	Pathological	Tolerated	Neutral	Neutral	Not afected	Not afected	Not afected	Slightly enhances
Glu21Gly	Disease	Probably damaging	Non neutral	Pathological	Tolerated	Disease	Disease	Not afected	Not afected	Not afected	Reduces
Gln22Leu	Disease	Benign	Non neutral	Pathological	Affect protein function	Disease	Disease	Not afected	Not afected	Not afected	Not afected
Gln22Arg	Disease	Probably damaging	Non neutral	Pathological	Affect protein function	Disease	Disease	Not afected	Not afected	Not afected	Reduces
Val29Ala	Disease	Probably damaging	Non neutral	Neutral	Tolerated	Neutral	Disease	Not afected	Not afected	Not afected	Reduces
Gly37Arg	Disease	Probably damaging	Non neutral	Pathological	Affect protein function	Disease	Disease	Not afected	Not afected	Not afected	Severely reduces
Leu38Arg	Disease	Probably damaging	Non neutral	Pathological	Affect protein function	Disease	Disease	Not afected	Not afected	Not afected	Severely reduces
Leu38Val	Disease	Probably damaging	Non neutral	Neutral	Affect protein function	Disease	Neutral	Not afected	Not afected	Not afected	Reduces
Gly41Ser	Disease	Probably damaging	Non neutral	Pathological	Affect protein function	Disease	Disease	Not afected	Not afected	Not afected	Reduces
Gly41Asp	Disease	Probably damaging	Non neutral	Pathological	Affect protein function	Disease	Disease	Not afected	Not afected	Not afected	Reduces
His43Arg	Disease	Probably damaging	Non neutral	Pathological	Tolerated	Disease	Disease	Not afected	Not afected	Not afected	Reduces

Phe45Cys	Disease	Probably damaging	Non neutral	Pathological	Affect protein function	Disease	Disease	Not afected	Not afected	Not afected	Reduces
His46Arg	Disease	Probably damaging	Non neutral	Pathological	Affect protein function	Disease	Disease	Not afected	Not afected	Not afected	Severely reduces
Val47Phe	Disease	Probably damaging	Non neutral	Pathological	Affect protein function	Disease	Disease	Not afected	Not afected	Not afected	Slightly enhances
Val47Ala	Disease	Probably damaging	Non neutral	Neutral	Affect protein function	Disease	Disease	Not afected	Not afected	Not afected	Reduces
His48Arg	Disease	Probably damaging	Non neutral	Pathological	Affect protein function	Disease	Disease	Not afected	Not afected	Not afected	Reduces
His48Gln	Disease	Probably damaging	Non neutral	Pathological	Affect protein function	Disease	Disease	Not afected	Not afected	Not afected	Reduces
Glu49Lys	Disease	Benign	Non neutral	Neutral	Tolerated	Disease	Neutral	Not afected	Not afected	Not afected	Slightly reduces
Thr54Arg	Disease	Benign	Non neutral	Pathological	Affect protein function	Disease	Neutral	Not afected	Not afected	Not afected	Not afected
Ser59Ile	Disease	Probably damaging	Non neutral	Pathological	Affect protein function	Disease	Neutral	Not afected	Not afected	Not afected	Reduces
Gly61Arg	Disease	Probably damaging	Non neutral	Pathological	Affect protein function	Disease	Disease	Not afected	Not afected	Not afected	Reduces
Asn65Ser	Disease	Probably damaging	Non neutral	Neutral	Affect protein function	Disease	Neutral	Not afected	Not afected	Not afected	Not afected
Pro66Ala	Disease	Probably damaging	Non neutral	Neutral	Affect protein function	Neutral	Neutral	Not afected	Not afected	Not afected	Reduces
Pro66Ser	Disease	Probably damaging	Non neutral	Neutral	Affect protein function	Disease	Neutral	Not afected	Not afected	Not afected	Reduces
Leu67Arg	Disease	Benign	Non neutral	Neutral	Tolerated	Disease	Neutral	Not afected	Not afected	Not afected	Not afected
Gly72Ser	Disease	Probably damaging	Non neutral	Pathological	Affect protein function	Disease	Disease	Not afected	Not afected	Not afected	Reduces
Gly72Cys	Disease	Probably damaging	Non neutral	Pathological	Affect protein function	Disease	Disease	Not afected	Not afected	Not afected	Reduces
Asp76Val	Disease	Probably damaging	Non neutral	Neutral	Affect protein function	Disease	Neutral	Not afected	Not afected	Not afected	Slightly reduces

Asp76Tyr	Disease	Probably damaging	Non neutral	Pathological	Affect protein function	Disease	Neutral	Not afected	Not afected	Not afected	Not afected
His80Arg	Disease	Probably damaging	Non neutral	Pathological	Affect protein function	Disease	Disease	Not afected	Not afected	Not afected	Severely reduces
His80Ala	Disease	Probably damaging	Non neutral	Pathological	Affect protein function	Disease	Disease	Not afected	Not afected	Not afected	Reduces
Leu84Phe	Disease	Probably damaging	Non neutral	Pathological	Affect protein function	Disease	Disease	Not afected	Not afected	Not afected	Reduces
Leu84Val	Disease	Probably damaging	Non neutral	Neutral	Affect protein function	Disease	Neutral	Not afected	Not afected	Not afected	Reduces
Gly85Arg	Disease	Probably damaging	Non neutral	Pathological	Affect protein function	Disease	Disease	Not afected	Not afected	Not afected	Severely reduces
Gly85Ser	Disease	Probably damaging	Non neutral	Pathological	Affect protein function	Disease	Disease	Not afected	Not afected	Not afected	Reduces
Asn86Ser	Disease	Probably damaging	Non neutral	Neutral	Affect protein function	Disease	Disease	Not afected	Not afected	Not afected	Reduces
Asn86Asp	Disease	Probably damaging	Non neutral	Neutral	Affect protein function	Disease	Neutral	Not afected	Not afected	Not afected	Reduces
Asn86Lys	Disease	Probably damaging	Non neutral	Pathological	Affect protein function	Disease	Neutral	Not afected	Not afected	Not afected	Not afected
Asn86Ile	Disease	Probably damaging	Non neutral	Pathological	Affect protein function	Disease	Disease	Not afected	Not afected	Not afected	Not afected
Val87Met	Disease	Probably damaging	Non neutral	Neutral	Affect protein function	Disease	Neutral	Not afected	Not afected	Not afected	Slightly reduces
Val87Ala	Disease	Probably damaging	Non neutral	Neutral	Affect protein function	Disease	Disease	Not afected	Not afected	Not afected	Reduces
Ala89Thr	Disease	Probably damaging	Non neutral	Neutral	Tolerated	Disease	Neutral	Not afected	Not afected	Not afected	Reduces
Ala89Val	Disease	Probably damaging	Non neutral	Pathological	Tolerated	Disease	Neutral	Not afected	Not afected	Not afected	Severely reduces
Asp90Val	Disease	Benign	Non neutral	Pathological	Affect protein function	Disease	Neutral	Not afected	Not afected	Not afected	Reduces
Asp90Ala	Disease	Benign	Non neutral	Pathological	Tolerated	Disease	Neutral	Not afected	Not afected	Not afected	Reduces

Gly93Val	Disease	Probably damaging	Non neutral	Neutral	Affect protein function	Disease	Disease	Not afected	Not afected	Not afected	Severely reduces
Gly93Ala	Disease	Probably damaging	Non neutral	Neutral	Affect protein function	Disease	Disease	Not afected	Not afected	Not afected	Reduces
Gly93Cys	Disease	Probably damaging	Non neutral	Pathological	Affect protein function	Disease	Disease	Not afected	Not afected	Not afected	Reduces
Gly93Arg	Disease	Probably damaging	Non neutral	Pathological	Affect protein function	Disease	Disease	Not afected	Not afected	Not afected	Reduces
Gly93Asp	Disease	Probably damaging	Non neutral	Pathological	Affect protein function	Disease	Disease	Not afected	Not afected	Not afected	Severely reduces
Gly93Ser	Disease	Probably damaging	Non neutral	Pathological	Affect protein function	Disease	Disease	Not afected	Not afected	Not afected	Reduces
Ala95Val	Disease	Probably damaging	Non neutral	Pathological	Tolerated	Disease	Neutral	Not afected	Not afected	Not afected	Reduces
Ala95Thr	Disease	Probably damaging	Non neutral	Neutral	Tolerated	Disease	Neutral	Not afected	Not afected	Not afected	Reduces
Ala95Gly	Disease	Probably damaging	Non neutral	Neutral	Affect protein function	Disease	Disease	Not afected	Not afected	Not afected	Reduces
Asp96Val	Disease	Benign	Neutral	Neutral	Tolerated	Disease	Neutral	Increases	Not afected	Not afected	Not afected
Asp96Asn	Disease	Benign	Neutral	Neutral	Tolerated	Neutral	Neutral	Not afected	Not afected	Not afected	Not afected
Val97Met	Disease	Probably damaging	Non neutral	Neutral	Tolerated	Neutral	Neutral	Not afected	Not afected	Not afected	Reduces
Val97Leu	Disease	Probably damaging	Non neutral	Pathological	Tolerated	Neutral	Neutral	Not afected	Not afected	Not afected	Reduces
Ile99Val	Disease	Benign	Neutral	Neutral	Tolerated	Neutral	Neutral	Not afected	Not afected	Not afected	Reduces
Glu100Gly	Disease	Benign	Non neutral	Pathological	Tolerated	Disease	Neutral	Not afected	Not afected	Not afected	Slightly reduces
Glu100Lys	Disease	Benign	Non neutral	Neutral	Tolerated	Neutral	Neutral	Not afected	Not afected	Not afected	Not afected

Asp101Gly	Disease	Probably damaging	Non neutral	Pathological	Affect protein function	Disease	Disease	Not afected	Not afected	Not afected	Severely reduces
Asp101Asn	Disease	Probably damaging	Non neutral	Neutral	Affect protein function	Disease	Neutral	Not afected	Not afected	Not afected	Reduces
Asp101Tyr	Disease	Probably damaging	Non neutral	Pathological	Affect protein function	Disease	Disease	Not afected	Not afected	Not afected	Severely reduces
Asp101His	Disease	Probably damaging	Non neutral	Neutral	Affect protein function	Disease	Neutral	Not afected	Not afected	Not afected	Severely reduces
Ile104Phe	Disease	Probably damaging	Non neutral	Pathological	Affect protein function	Disease	Disease	Not afected	Not afected	Not afected	Severely reduces
Ser105Leu	Disease	Probably damaging	Non neutral	Neutral	Tolerated	Disease	Neutral	Not afected	Not afected	Not afected	Reduces
Leu106Val	Disease	Probably damaging	Non neutral	Neutral	Affect protein function	Disease	Neutral	Not afected	Not afected	Not afected	Reduces
Leu106Phe	Disease	Probably damaging	Non neutral	Neutral	Affect protein function	Disease	Disease	Not afected	Not afected	Not afected	Severely reduces
Gly108Val	Disease	Probably damaging	Non neutral	Pathological	Affect protein function	Disease	Disease	Not afected	Not afected	Not afected	Severely reduces
Cys111Tyr	Disease	Benign	Non neutral	Pathological	Affect protein function	Disease	Unknown	Not afected	Not afected	Not afected	Reduces
Ile112Thr	Disease	Probably damaging	Non neutral	Pathological	Affect protein function	Disease	Disease	Not afected	Not afected	Not afected	Reduces
Ile112Met	Disease	Probably damaging	Non neutral	Neutral	Affect protein function	Disease	Neutral	Not afected	Not afected	Not afected	Reduces
Ile113Thr	Disease	Probably damaging	Non neutral	Pathological	Affect protein function	Disease	Neutral	Not afected	Not afected	Not afected	Reduces
Ile113Phe	Disease	Probably damaging	Non neutral	Neutral	Affect protein function	Disease	Neutral	Not afected	Not afected	Not afected	Reduces
Gly114Ala	Disease	Probably damaging	Non neutral	Neutral	Affect protein function	Disease	Disease	Not afected	Not afected	Not afected	Reduces
Arg115Gly	Disease	Probably damaging	Non neutral	Pathological	Affect protein function	Disease	Disease	Increases	Not afected	Not afected	Slightly reduces
Leu117Val	Disease	Benign	Neutral	Neutral	Tolerated	Neutral	Neutral	Not afected	Not afected	Not afected	Reduces

Val118Leu	Disease	Probably damaging	Non neutral	Pathological	Affect protein function	Disease	Neutral	Not afected	Not afected	Not afected	Reduces
Asp124Val	Disease	Probably damaging	Non neutral	Pathological	Affect protein function	Disease	Disease	Not afected	Not afected	Not afected	Reduces
Asp124Gly	Disease	Probably damaging	Non neutral	Pathological	Affect protein function	Disease	Disease	Not afected	Not afected	Not afected	Reduces
Asp125His	Disease	Probably damaging	Non neutral	Pathological	Affect protein function	Disease	Neutral	Not afected	Not afected	Not afected	Not afected
Leu126Ser	Disease	Probably damaging	Non neutral	Neutral	Affect protein function	Disease	Neutral	Not afected	Not afected	Not afected	Reduces
Gly127Arg	Disease	Probably damaging	Neutral	Pathological	Affect protein function	Disease	Disease	Not afected	Not afected	Not afected	Severely reduces
Glu133Val	Disease	Probably damaging	Non neutral	Pathological	Affect protein function	Disease	Unknown	Not afected	Not afected	Not afected	Slightly reduces
Ser134Asn	Disease	Probably damaging	Non neutral	Neutral	Affect protein function	Disease	Neutral	Not afected	Not afected	Not afected	Severely reduces
Thr137Arg	Disease	Probably damaging	Non neutral	Pathological	Affect protein function	Disease	Neutral	Not afected	Not afected	Not afected	Slightlys enhances
Gly138Glu	Disease	Probably damaging	Non neutral	Pathological	Affect protein function	Disease	Disease	Not afected	Not afected	Not afected	Severely reduces
Asn139His	Disease	Probably damaging	Non neutral	Neutral	Affect protein function	Disease	Neutral	Not afected	Not afected	Not afected	Reduces
Asn139Lys	Disease	Probably damaging	Non neutral	Pathological	Affect protein function	Disease	Neutral	Not afected	Not afected	Not afected	Slightly reduces
Asn139Asp	Disease	Probably damaging	Non neutral	Neutral	Affect protein function	Disease	Neutral	Not afected	Not afected	Not afected	Reduces
Ala140Gly	Disease	Probably damaging	Non neutral	Neutral	Affect protein function	Disease	Neutral	Not afected	Not afected	Not afected	Slightly reduces
Gly141Glu	Disease	Probably damaging	Non neutral	Pathological	Affect protein function	Disease	Disease	Not afected	Not afected	Not afected	Severely reduces
Leu144Phe	Disease	Probably damaging	Non neutral	Neutral	Tolerated	Disease	Disease	Not afected	Not afected	Not afected	Slightly reduces
Leu144Ser	Disease	Probably damaging	Non neutral	Pathological	Tolerated	Disease	Disease	Not afected	Not afected	Not afected	Reduces

Ala145Thr	Disease	Probably damaging	Non neutral	Pathological	Affect protein function	Disease	Neutral	Not afected	Not afected	Not afected	Severely reduces
Ala145Gly	Disease	Probably damaging	Non neutral	Neutral	Tolerated	Disease	Disease	Not afected	Not afected	Not afected	Reduces
Cys146Arg	Disease	Probably damaging	Non neutral	Pathological	Affect protein function	Disease	Disease	Increases	Not afected	Not afected	Severely reduces
Gly147Asp	Disease	Probably damaging	Non neutral	Pathological	Affect protein function	Disease	Disease	Decreases	Not afected	Not afected	Severely reduces
Gly147Arg	Disease	Probably damaging	Non neutral	Pathological	Affect protein function	Disease	Disease	Increases	Not afected	Not afected	Severely reduces
Val148Gly	Disease	Probably damaging	Non neutral	Neutral	Affect protein function	Disease	Neutral	Decreases	Not afected	Not afected	Not afected
Val148Ile	Disease	Probably damaging	Non neutral	Neutral	Tolerated	Neutral	Neutral	Not afected	Not afected	Not afected	Slightly enhances
Ile149Thr	Disease	Probably damaging	Non neutral	Pathological	Affect protein function	Disease	Disease	Decreases	Not afected	Not afected	Reduces
Ile151Thr	Disease	Probably damaging	Non neutral	Pathological	Affect protein function	Disease	Neutral	Decreases	Not afected	Not afected	Slightly reduces
Ile151Ser	Disease	Probably damaging	Non neutral	Pathological	Affect protein function	Disease	Neutral	Decreases	Not afected	Not afected	Reduces
